# Epigenetic Regulation and its Effects on Aging and Cardiovascular Disease

**DOI:** 10.7759/cureus.39395

**Published:** 2023-05-23

**Authors:** Khalid Sawalha, Nicholas Norgard, Angel López-Candales

**Affiliations:** 1 Cardiometabolic Diseases, Truman Medical Centers - University of Missouri Kansas City, Kansas City, USA; 2 Pharmacology and Therapeutics, Truman Medical Centers - University of Missouri Kansas City, Kansas City, USA; 3 Cardiovascular Medicine, University of Missouri Kansas City, Kansas City, USA

**Keywords:** health aging, cellular aging, : enviornmental monitoring, enviornmental, genetic editing, cardiovascular prevention, epigenetic mutations

## Abstract

Cardiovascular disease (CVD), specifically coronary atherosclerosis, is regulated by an interplay between genetic and lifestyle factors. Most recently, a factor getting much attention is the role epigenetics play in atherosclerosis; particularly the development of coronary artery disease. Furthermore, it is important to understand the intricate interaction between the environment and each individual genetic material and how this interaction affects gene expression and consequently influences the development of atherosclerosis. Our main goal is to discuss epigenetic regulations; particularly, the factors contributing to coronary atherosclerosis and their role in aging and longevity. We reviewed the current literature and provided a simplified yet structured and reasonable appraisal of this topic. This role has also been recently linked to longevity and aging. Epigenetic regulations (modifications) whether through histone modifications or DNA or RNA methylation have been shown to be regulated by environmental factors such as social stress, smoking, chemical contaminants, and diet. These sensitive interactions are further aggravated by racial health disparities that ultimately impact cardiovascular disease outcomes through epigenetic interactions. Certainly, limiting our exposure to such causative events at younger ages seems our “golden opportunity” to tackle the incidence of coronary atherosclerosis and probably the answer to longevity.

## Introduction and background

The overall impact on global morbidity and mortality has shifted from mainly infectious etiologies to a distinct landscape of predominantly chronic illnesses [1, 2]. Of these ailments, cardiovascular diseases (CVD) continue as the leading cause worldwide [3]. As far as numerical estimates, CVD deaths were responsible for 14.4 million global deaths in 1990; a number that increased to 17.5 million by 2005 and was last tallied at approximately 17.9 million in 2019 [4].

Even though a series of well-known risk factors have been linked to the development of CVD, global mortality rates have shown regional differences [5]. These differences have been closely analyzed by investigators participating in the Global Burden of Disease (GBD) study [6]. Data from this ongoing multinational collaboration study have provided comparable and consistent estimates of population health, incidence, prevalence, case fatality, mortality, and health risks from 204 countries and territories since 1990 [6]. The main goal of this GBD study is not only to track incidences but most importantly, to benchmark progress regarding efforts to reduce the CVD burden [6]. Unfortunately, it has become apparent that a large gap exists between our current understanding of risks and the ultimate development of CVD.

Traditionally we have attributed risk factors such as age, gender, presence of hypertension, dyslipidemia, diabetes mellitus, obesity, family history, and unhealthy behaviors including physical inactivity, poor dietary habits, and cigarette smoking with the development of CVD [7-9]. Useful clinical risk calculators have been devised based on each individual risk profile to estimate a 10-year Atherosclerotic Cardiovascular Disease (ASCVD) risk score for each patient. These ASCVD risk scores easily obtained during a clinical encounter have helped practitioners establish a reference point with the intention not only of forecasting the risk of developing CVD but also, the potential impact of different interventions to minimize this risk [10].

In addition, the use of cardiac biomarkers such as B-type natriuretic peptide, troponin, soluble thrombomodulin 2, galectin-3, lipoprotein(a), homocysteine, fibroblast growth factor 23, adiponectin, glycated hemoglobin, haptoglobin, although less useful than initially intended, are also available to improve CVD risk stratification in certain patients [11, 12].

To improve individual ASCVD risk stratification, the potential impact of environmental stressors has been considered as a possible contributor to CVD [13]. In fact, data from the Prospective Urban Rural Epidemiology (PURE) study has helped in recognizing the complex interplay that exists between genetics and lifestyle factors in modulating individual expression of CVD [14].

These findings should ignite our interest regarding the potential interaction between external forces such as our individual behaviors and the environment we live as plausible elements that might affect our system and the risk each of us has in developing any given disease, based on our pre-specified genetic makeup.

Consequently, the study of epigenetics as it pertains to CVD should be a very close field of interest. Unlike pre-determined genetic changes within our genome that control gene expression; epigenetics study how external forces regulate or alter our genetic expression without causing any irreversible change to our genetic DNA sequence.

**Figure 1:**
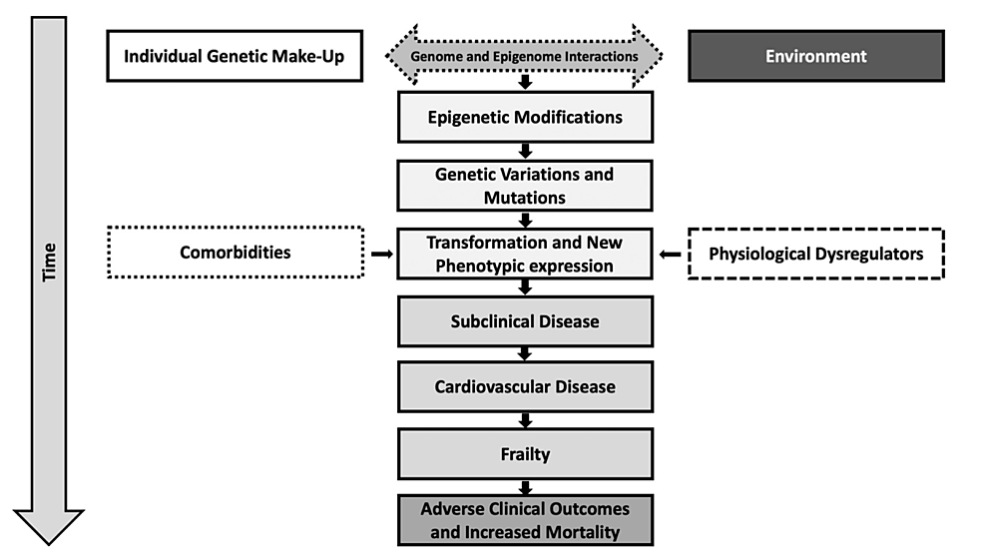
Algorithm showing the relationship between the environment and modulation of gene expression that is ultimately responsible of the phenotypic manifestation of CVD. Authors' own work. No permission needed.

Even though epigenetics is a term known for over a century ago; the use of this term facilitated by current technology has become more relevant and might be the necessary venue we need to close the gap and gain a better understanding of how the environment modulates gene expression and is ultimately responsible for different phenotypic expressions of CVD as shown in Figure 1.

**Figure 2:**
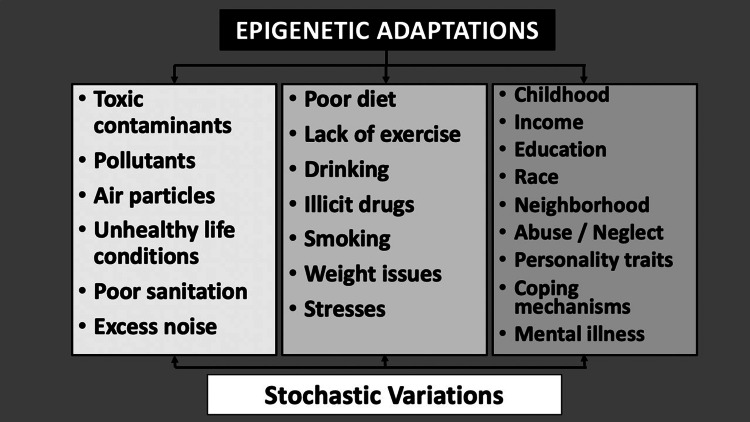
Showing different environmental factors that affect our genetic expression through epigenetic modifications and stochastic variations, ultimately responsible for abnormal phenotypic CVD manifestations. Authors' own work and does not require permission.

Now that we have the technical ability, it is crucial to advance our knowledge of how environmental factors modulate the expression of CVD. This would be particularly useful as through epigenetics; the importance of nutrition, smoking, pollution, stress, noise, allostatic factors, and alterations of the circadian rhythm have been already linked to a wide range of changes in gene expression (Figure 2) [15]. 

The crucial relevance of epigenetic studies relies on the strong association that the environment exerts on our genome resulting in changes in gene expression without permanent changes in the individual DNA sequence [16]. On that note, let’s recall some of the basic concepts that we learned in biology. Every nucleated cell in the body contains the same DNA sequence, which carries the organism's entire genome and includes coding and non-coding regions of DNA [17]. Despite having the same genetic code, cells from different tissues behave differently. These differences are not explained by differences in genome sequence but rather are created by additional layers of regulation that determine which genes are expressed to create a cell-specific phenotype. Epigenetic regulation is a chemical alteration to our DNA that can increase or decrease the expression of our genes without altering the genomic sequence, contributing to this cell-type-specific variation. Unlike changes to the genomic sequence, epigenetic changes are reversible [18].

## Review

**Figure 3:**
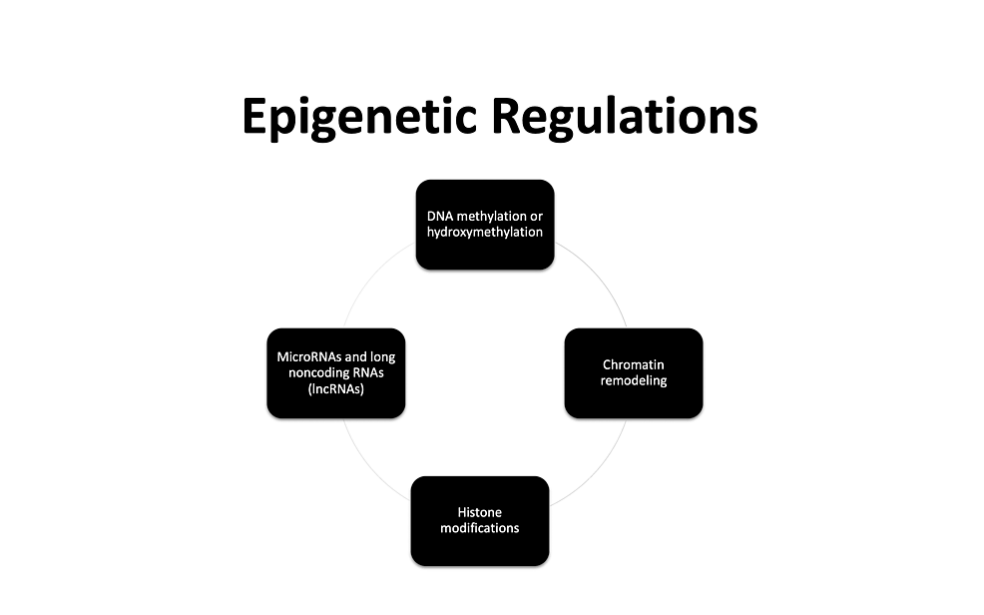
Showing several epigenetic mechanisms including DNA methylation or hydroxymethylation, histone modifications/variants, chromatin remodeling, microRNAs (miRNAs), and long noncoding RNAs (lncRNAs) that are relevant to the study of different phenotypic expressions of cardiovascular disease.

Even though several epigenetic mechanisms including DNA methylation or hydroxymethylation, histone modifications, chromatin remodeling, histone variants, microRNAs (miRNAs), and long noncoding RNAs (lncRNAs) are relevant to the study of different phenotypic expressions of CVD [19-21], we would like to simplify terms and mainly describe two main forms of epigenetic regulations (Figure 3).

First is DNA methylation which involves the addition of a methyl group (CH3) to carbon 5 in a cytosine base to create 5-methylcytosine. It is almost exclusively occurring in cytosines. Common sites for DNA methylation include promoter or enhancer regions of the affected gene which are called cis-regulatory elements. The enzymes that catalyze DNA methylation are referred to as DNA methyltransferases. The process of adding more methyl groups is referred to as DNA hypermethylation while the removal of one or more methyl groups from the cytosine bases is called DNA hypomethylation [22].

The second is histone modifications. Histones are proteins that form a multi-subunit core in which DNA can be wrapped to form a nucleosome; this is the first level of DNA compaction needed to assemble linear DNA into highly compacted chromosomes [23]. Several histone modifications have been described including methylation and acetylation, among others [24]. Histone methylation is the addition of a methyl group to the amino acid lysine at the histone tail, the enzyme that catalyzes this process is known as histone methyltransferases, and the enzymes that remove these methyl groups are referred to as histone demethylases. Histone acetylation refers to the addition of an acetyl group (CH3CO) to lysine in a histone protein [25]. The enzymes that catalyze histone acetylation are referred to as histone acetyltransferases and that catalyze the removal of an acetyl group from a histone are called histone deacetylases [26].

Now we have this fundamental knowledge, let's dive into the potential value of epigenetics in developing CVD. However, we should direct our attention to the role of epigenetics in aging. The latter not only is clinically relevant in determining longevity but also, in the expression of CVD.

Therefore, let's first explore what role epigenetics play in both aging and longevity. 

Aging is defined as the time-related deterioration of physiological functions necessary for survival and fertility [27]. Deterioration of these essential functions places individuals at risk of diseases. Even though similar individuals might experience similar environmental exposures; their individual genetic makeup places them either at less or greater risk for developing any given particular disease. In contrast, our individual susceptibility to any given disease not only is determined by the individual genetic makeup but also, by multiple external factors such as our particular lifestyle choices and environmental exposure. To attest to this idea, the Danish twin study found that human genetics was responsible for up to 25% variation in longevity between twins [28]. Furthermore, these investigators identified environmental factors as responsible for affecting longevity in 50% of cases [28].

In addition, epigenetic DNA regulations affected by age have been shown to be highly tissue-specific [29]. In other words, upregulation of RNA transcripts and downregulation of critical metabolically important regulatory proteins mainly occur at mid-age while more significant basic cellular functions including DNA repair and oxidative phosphorylation are affected later in life, contributing to cellular decay and adverse events [29].

The term “successful aging” or expected normal longevity has been conceptualized as the “normal” temporal progression of the life of an individual not affected by any chronic disease or condition that becomes either damaging or debilitating. Consequently, these individuals continue to function independently into old age while remaining both physically and cognitively intact.

As already stated, the interplay of genetics and the environment play a significant role in longevity and life expectancy. Furthermore, results from Franceschi et al. using data from the Italian Multicenter Study on Centenarians (IMUSCE) were helpful in showing how epigenetics vary in accordance with gender [30]. These investigators found that the ratio between females and males among centenarians in Italy differs significantly [30]. Specifically, this ratio ranged from 2:1 in Sardinia to 7:1 among inhabitants from Northern Italy [30]. 

In another study, Willcox and associates [31] studied the effect of environmental exposure on longevity. These investigators found "caloric restriction” was a main determinant of longevity among participants living in Okinawa, Japan [31]. It is important to highlight the findings of this study as it provides epidemiologic support for the benefits of caloric restriction in humans [31]. These findings were in accordance with the well-known literature on animals with regard to caloric restriction and healthy aging [31].

To further test if early life events were associated with normal aging without disease, a British civil service study that followed participants for up to 17 years found that interventions meant to promote healthy behaviors were able to mitigate the overall harmful effects of other less-modifiable risk factors while reducing social inequalities [32]. However, a gender difference in longevity was noted [32]. Based on their data, investigators concluded that genetics was less important in determining the differences between males and females while differences concerning healthier lifestyles and favorable environmental conditions were more critically relevant [32].

As previously stated, “successful aging” implies that individuals function while physically and cognitively intact but are not afflicted by any chronic illness. These requirements were longitudinally evaluated in the Cardiovascular Health Study [33]. A total of 1677 participants (median age 85 years, ranging from 77 to 102 years) were prospectively followed for 14 years [33]. Upon determination of functional physical and cognitive status as well as behavioral health factors as well as the presence or absence of any chronic illness, functional decline was measured over time [33]. Importantly, participants with less functional decline not only had higher values at the onset of the study but also, a much better baseline health profile [33]. These investigators also found that women and participants with weight issues (overweight or obesity) had higher rates of physical impairment without impact on their cognitive abilities [33]. They also noted that the most common risk factors for the development of functional and cognitive decline were the presence of CVD and hypertension [33].

Before we truly assess the role of epigenetics in CVD; we believe it is important to first examine the role of epigenetics and health disparities. The latter has been defined as undesirable differences in health outcomes that are generally not driven by informed differences based on patient preferences [34]. These differences are concerning as they are prevalent in all spheres of health and disease [34]. They can arise from myriad contributors, including socioeconomic factors, education, and environment. These inequalities can include the patient’s race/ethnicity, sex, gender, religion, socioeconomic status, immigration status, and other characteristics [35].

Recent studies have supported the relationship between these health disparities and the development of CVD through epigenetics, particularly through changes in DNA methylation mediated by exposure to smoking, air pollution, alcohol, and stress [36]. This was further supported by Zhong et al., when they evaluated the toll-like receptor 2 (TLR2) methylation process, which is a reversible immune‐epigenetic activity, following exposure to the short‐term fine particles (PM2.5) in 573 elderly men from the normative aging study. In the group with higher TLR2 methylation rates a greater susceptibility to adverse cardiac autonomic is anticipated [37].

Would epigenetics helps us explain differences in morbidity and mortality among African Americans when considering CVD and cancer? [38]. Certainly, additional research is warranted to advance our understanding of how environmental and lifestyle factors mediate epigenetic modifications which might help decrease adverse CVD events and minimize disparities in health care. 

Now we should be able to get a better look at the role of epigenetics as it relates to atherosclerosis.

Vascular injury leading to unrelenting atherosclerotic injury is a well-described phenomenon [39]. A number of cellular events involved in the initial injury as well as the factors involved in the growth of vascular lesions have been well-characterized [40-43]. The intricate interaction of activated cellular elements in response to vascular injury and the abnormal disruption of cholesterol homeostasis coupled with the activation of oxidative metabolites and inflammatory cells characterize the process of atherosclerosis [40-43]. Although this atherosclerotic vascular injury is more common among diabetes; this process can happen independent of abnormal blood sugar metabolism. However, an abnormal diabetic environment accelerates vascular injury and worsens the inflammatory response and cellular damage [44, 45].

The pathophysiology of epigenetics in coronary atherosclerosis, specifically coronary artery disease is getting more attention and has sparked researchers to continue investigating this field to gain a better understanding of the events that unfold the progression of disease. Epigenetic regulations play a vital role in coronary atherosclerosis and are sensitive to environmental factors such as social stress, smoking, chemical contaminants, and diet [46]. 

Current data support the notion that extensive epigenetic changes occur at different levels of the atherosclerosis process such as endothelial cell proliferation, vascular smooth muscle differentiation, macrophage accumulation, and inflammatory pathway activation [47].

Therefore, careful analysis performed at a young age might help us limit damaging exposure that ultimately might alter our genomic expression to express adverse CVD phenotypes. This ability to identify culprits early on might not only be our “golden opportunity” to mitigate the effects of CVD but also, answer the question of how to attain healthier longevity.

With all this information it is now safe to consider the potential utility of epigenetics as a potentially useful alternative for future interventions regarding prevention of CVD.

The modifiable and reversible nature of epigenetic regulations sheds light on future therapies in cardiovascular disease, possibly using pharmaceutical agents or interventions. Recent studies have investigated the possibility of interventions aimed to reduce the ongoing inflammatory response aggravating vascular injury in atherosclerosis-specific agents such as DNA methyltransferase, histone acetyltransferase, histone deacetylase, and histone methylation inhibitors [48].

Finally, as Horvath once suggested, the use of “epigenetic clocks” might one day become useful in aging and cancer research while also being a valuable tool that can be used in the early identification of individuals at risk of developing CVD [49].

In this regard, epigenetics appear to be a promising field for future therapies and interventions to reduce CVD. Surely, the development of these applications might result in broader applications that can offer potentially greater benefits. We can only hope that the evolution of this field would ultimately improve human lives not only by reducing CVD but also giving us the opportunity to achieve healthier aging.

In this article we have provided a concise review of epigenetics; particularly, how the most common epigenetic regulations mediated by alterations from environmental exposure such as social stressors, smoking, chemical contaminants, and unhealthy diets alter either DNA or RNA and through these mechanisms contribute to vascular injury and the process of atherosclerosis. Similar external stressors appear to be responsible for altering our DNA through epigenetic interactions resulting in unhealthy aging and a shorter lifespan (Table 1). Certainly, limiting our exposure to such causative events at a younger age seems to be our “golden opportunity” in minimizing our overall risk of complications from atherosclerosis and improving our longevity.

The following table (Table 1) provides a review of the most important epigenetic manifestations and the proposed mechanisms responsible for each phenotypic expression [50-82]. 

**Table 1:** Descriptive analysis of the most recognizable epigenetic interactions and the proposed mechanisms through which each specific interaction is phenotypically manifested

Specific Epigenetic Interaction	Epigenetic Mechanism(s)
Aging [50-54]	Overall decrease in deoxyribonucleic acid (DNA) methylation with increasing age telomere shortening results in cell division slowing down leads to accelerated cell senescence and a higher risk for developing age-related diseases
Obesity [54-59]	Increased body mass index is associated with alterations of DNA methylation levels at several cytosine and guanine bases connected by a phosphate group (CpGs) within metabolic genes including the hypoxia-inducible factor alpha gene (HIF3A), insulin-like growth factor binding protein (IGFBP3), autophagy and sterol regulatory element binding proteins (SREBF1), tumor necrosis factor (TNF), tripartite motif containing 3 (TRIM3) and ubiquitin associated and SH3 domain containing A (UBASH3A) as well as global methylation levels of Alu–elements genes or "jumping gene" or transposable element. Senescence-relevant genes hypomethylation such as p21, p16 as well as the telomerase reverse transcriptase (TERT)’s hypermethylation accelerate adipose progenitor cellular senescence and exhaustion Increase in methylation levels of the regulator regions in peroxisome proliferator-activated receptor gamma (Ppar-γ). Adiponectin (ADIPOQ) and leptin (LEP) have been associated with white adipocyte glucose and lipid metabolism alterations, which further worsen insulin resistance, obesity, and inflammation. Hypermethylation of the PR-domain containing 16 protein (PRDM16) and the enhancer region of uncoupling protein-1 (UCP1) during cellular senescence, triggers to loss of beige adipose tissue and thermogenic properties of brown adipocytes
Inflammation [60-64]	Activation of the p65 subunit promoter of the nuclear factor kappa (NF-κB)-light-chain-enhancer of activated B cells. Increase in the histone acetyltransferases (HATs) activity. Hyperacetylation of inflammation-related genes promoters such as tumor necrosis factor-α (TNF-α) and cyclooxygenase-2. Regulation of pro-inflammatory genes MicroRNAs
Atherosclerosis [65-68]	Hypermethylation of atheroprotective estrogen receptor α (ESR1) and estrogen receptor β (ESR2) in vascular smooth muscle cells Hypomethylation of the promoter of coagulation factor VII was associated with coronary artery disease microRNAs
Diabetic vascular disease [69, 70]	Hyperglycemia induces specific chromatin changes and transcriptional responses as well as histone modifications and DNA methylation
Smoking [71]	DNA methylation
Cardiac hypertrophy [72-74]	Histone acetylation implicating both histone acetyltransferases (HATs) and histone deacetylases (HDACs) microRNAs
Heart failure [75-78]	Three angiogenesis-related genes were differentially methylated, irrespective of the etiology: angiomotin-like 2 gene (AMOTL2) was hypomethylated whereas the 5′ promoter region in the platelet/endothelial cell adhesion molecule gene (PECAM1) and the gene body of Rho GTPase-activating protein 24 (ARHGAP24) were hypermethylated Histone modifications are also important; particularly, genome-wide histone methylation of heart tissues Tri-methylated histone H3H4 and H3K9 micro-RNAs
Arrhythmias [79-82]	Specific HDAC inhibitor reverses atrial fibrosis and diminishes atrial fibrillation vulnerability following electrical stimulation. Micro-RNAs (miR-1) are essential in normal electrophysiological conduction and their deletion is associated with a high rate of sudden death Overexpression of miR-208a is also associated with arrhythmia and heart death Increases in miR-133a leads to prolonged QT intervals. MicroRNAs miR-212, miR-17-92, miR-155, miR-181, and miR-181a have been associated with the regulation of heart rhythm through the regulation of ion channels, transporters, and cellular proteins

## Conclusions

Epigenetics has influenced our understanding of gene regulations in cardiovascular disease, aging, and longevity, but further work in this aspect is needed to provide the opportunity to develop strategies for prevention, early diagnosis, or even treatment of the same. Although the future might appear brighter as we have uncovered these truths regarding the interaction of our genetic makeup with the environment, much more research is needed. Not all available data have yielded consistent results and the full impact of environmental stressors on specific demographic groups might yield different results. Furthermore, potential eventual interventions might not be applicable to everyone and treatment responses might vary at different stages of our life. In essence, more rigorous studies are certainly needed so we can improve our understanding of epigenetics not only in regulating CVD but also aging and longevity.
